# Are women with major depression in pregnancy identifiable in population health data?

**DOI:** 10.1186/1471-2393-13-63

**Published:** 2013-03-12

**Authors:** Lyn Colvin, Linda Slack-Smith, Fiona J Stanley, Carol Bower

**Affiliations:** 1 Telethon Institute for Child Health Research, Centre for Child Health Research, The University of Western Australia, Perth, Australia; 2 School of Dentistry, The University of Western Australia, Perth, Australia; 3 Western Australian Register of Developmental Anomalies, Perth, Australia

**Keywords:** Population-based, Data linkage, Pharmacovigilance, Case ascertainment, Depression, Pregnancy, Antidepressant

## Abstract

**Background:**

Although record linkage of routinely collected health datasets is a valuable research resource, most datasets are established for administrative purposes and not for health outcomes research. In order for meaningful results to be extrapolated to specific populations, the limitations of the data and linkage methodology need to be investigated and clarified. It is the objective of this study to investigate the differences in ascertainment which may arise between a hospital admission dataset and a dispensing claims dataset, using major depression in pregnancy as an example. The safe use of antidepressants in pregnancy is an ongoing issue for clinicians with around 10% of pregnant women suffer from depression. As the birth admission will be the first admission to hospital during their pregnancy for most women, their use of antidepressants, or their depressive condition, may not be revealed to the attending hospital clinicians. This may result in adverse outcomes for the mother and infant.

**Methods:**

Population-based de-identified data were provided from the Western Australian Data Linkage System linking the administrative health records of women with a delivery to related records from the Midwives’ Notification System, the Hospital Morbidity Data System and the national Pharmaceutical Benefits Scheme dataset. The women with depression during their pregnancy were ascertained in two ways: women with dispensing records relating to dispensed antidepressant medicines with an WHO ATC code to the 3rd level, pharmacological subgroup, ‘N06A Antidepressants’; and, women with any hospital admission during pregnancy, including the birth admission, if a comorbidity was recorded relating to depression.

**Results:**

From 2002 to 2005, there were 96698 births in WA. At least one antidepressant was dispensed to 4485 (4.6%) pregnant women. There were 3010 (3.1%) women with a comorbidity related to depression recorded on their delivery admission, or other admission to hospital during pregnancy. There were a total of 7495 pregnancies identified by either set of records. Using data linkage, we determined that these records represented 6596 individual pregnancies. Only 899 pregnancies were found in both groups (13.6% of all cases). 80% of women dispensed an antidepressant did not have depression recorded as a comorbidity on their hospital records. A simple capture-recapture calculation suggests the prevalence of depression in this population of pregnant women to be around 16%.

**Conclusion:**

No single data source is likely to provide a complete health profile for an individual. For women with depression in pregnancy and dispensed antidepressants, the hospital admission data do not adequately capture all cases.

## Background

Data linkage of administrative data has been a rich resource for Western Australian researchers for a number of years [1-5]. The more recent approval to link national data from the Pharmaceutical Benefits Scheme (PBS) to datasets in the Western Australian Data Linkage System (WADLS) provides new and valuable opportunities to examine birth outcome profiles of prescription medicines dispensed for use during the preconception period and pregnancy. However, most of the datasets were established for administrative purposes and not for health outcomes research. In order for meaningful results to be extrapolated to specific populations, the limitations of the data and linkage methodology need to be investigated and clarified. An important step is to understand the limits of case ascertainment within each dataset.

The Mental Health Services in Australia report uses data from the National Survey of Mental Health and Wellbeing 2007 (N = 8800 Australians aged 16–85 years), and from the National Mental Health Establishments Database of the Australian Institute of Health and Wellbeing [6]. In the foreword to the 2006–2007 report, the director notes, “At this stage of data development, the information we can provide is limited to the number of services, or visits, or prescriptions delivered across Australia. We still have very little information about the number of people involved, or the services used per person. This remains an important data gap that can only be addressed by connecting information for mental health consumers within and across various datasets. This might be achieved by a range of strategies including data linkage as well as the information that might flow from the implementation of e-health” [7]. The report showed the most common type of management reported for mental health-related problems was a medication being prescribed, supplied or recommended by the general practitioner. Antidepressants were the most common medication, followed by anxiolytics, and hypnotics and sedatives.

Depression is a major public health issue in Australia. In 2009–10, more than 10% of all PBS prescription claims were related to mental health conditions [8]. A large Australian study found that around 9% of women experienced depression in the antenatal period and 16% in the postnatal period, [9] so depression, as an identified condition, should be well-represented in the administrative health datasets relating to pregnancy.

It is the objective of this study to investigate the differences in ascertainment between two datasets, using major depression in pregnancy as an example.

## Methods

This was a population-based data linkage study investigating pregnancy events in WA from 2002 to 2005. A pregnancy event was defined as a hospital admission record in the Hospital Morbidity Data System (HMDS) with a diagnosis code between O00-O99, based upon the International Statistical Classification of Diseases and Related Health Problems, Tenth Revision, Australian Modification (ICD-10-AM) [10]. De-identified data were provided from the WADLS, linking the records of women with each pregnancy event to any related records in the HMDS, the Midwives’ Notification System (MNS), and the Registry of Births and Deaths. These datasets were linked to each other and to data from the national PBS. The linkages and methodology have been described previously [11,12].

With data linkage, we could overlay the dates of each woman’s pregnancy from the MNS (based upon last menstrual period and delivery date) to the PBS dispenses to each woman within the same time frame, to determine exposures to PBS medicines during pregnancy. Using these dates we also determined the hospital admissions for each woman that occurred during her pregnancy. There were 112 pregnancies without an MNS record and these were excluded in the initial validation of the datasets.

In Australia, community prescriptions (i.e. non-public hospital) are dispensed either as private prescriptions or under one of two subsidisation schemes—the PBS and the Repatriation Pharmaceutical Benefits Scheme. All Australians are eligible to receive subsidised rates for prescribed medicines approved under the PBS, with around 80% of prescriptions dispensed in Australia being subsidised. Patients are grouped into two classes: general and concessional. As the general patient copayment rises, the dispensed prices of many of the cheaper medications fall under this level. In such cases the patient pays the full price and no claim for payment is made under the PBS. For the year ending June 2006, 83.8% of all dispenses and 80.0% of expenditure recorded on the PBS were for concessional patients [13]. New medicines are usually listed on the PBS at the full copayment amount, and hence all dispenses are captured in the data. However, the cost to the patient of older medicines and generic versions tend to fall below the copayment level and so not all medicines have been recorded previously for general patients.

Records from the PBS relating to antidepressant use were ascertained by selecting those dispenses of medicines with a WHO Anatomical Therapeutic Chemical (ATC) code to the 3rd level, pharmacological subgroup, ‘N06A Antidepressants.’ Women with any dispense of a medicine during her pregnancy that included one of these codes were ascertained as a PBS case. The medicines in this group are listed in Table 1. The copayment amount for general patients for fluvoxamine maleate 50 mg tablets and moclobemide 150 mg tablets fell below the subsidy level from April to December 2005 and from August to December 2005 for fluoxetine hydrochloride 20 mg tablets. Other forms of fluvoxamine maleate and moclobemide were listed at the maximum copayment level during this time. All other antidepressants were listed at the maximum copayment level for the period of the study. The PBS did not collect data on dispenses to patients in public hospitals until December 2004. As depression is a chronic condition, it is not likely that a woman would be dispensed an antidepressant only once and whilst she was in hospital.

**Table 1:** Ascertainment of pregnant women with depression from dispensing data, 2002-2005

	**Pregnancy Risk Code**	**Dispenses**		**PBS cases**		**PBS, no HMDS**		**PBS and HMDS**	
		**N**	**%**	**N**	**%**	**N**	**%**	**N**	**%**
**Pharmaceutical Benefits Scheme**		**20879**	**100.0%**	**4485**	**100.0%**	**3586**	**80.0%**	**899**	**20.0%**
Sertraline hydrochloride	C	5536	26.5%	1340	29.9%	1082	30.2%	258	28.7%
Citalopram hydrobromide	C	4784	22.9%	1136	25.3%	897	25.0%	239	26.6%
Paroxetine hydrochloride*	C/D	3180	15.2%	676	15.1%	565	15.8%	111	12.3%
Venlafaxine hydrochloride	B2	3146	15.1%	581	13.0%	430	12.0%	142	15.8%
Fluoxetine hydrochloride	C	1509	7.2%	364	8.1%	280	7.8%	84	9.3%
Escitalopram oxalate	C	775	3.7%	241	5.4%	189	5.3%	52	5.8%
Mirtazapine	B3	510	2.4%	140	3.1%	85	2.4%	55	6.1%
Fluvoxamine maleate	C	412	2.0%	140	3.1%	115	3.2%	25	2.8%
Amitriptyline hydrochlorine	C	273	1.3%	105	2.3%	79	2.2%	26	2.9%
Dothiepin hydrochloride	C	376	1.8%	78	1.7%	62	1.7%	16	1.8%
Moclobemide	B3	92	0.4%	40	0.9%	34	0.9%	6	0.7%
Doxepine hydrochloride	C	137	0.7%	32	0.7%	20	0.6%	12	1.3%
Reboxetine mesilate	B1	89	0.4%	24	0.5%	16	0.4%	8	0.9%
Imipramine hydrochloride	C	31	0.1%	8	0.2%	6	0.2%	<5	0.2%
Mianserin hydrochloride	B2	9	0.0%	6	0.1%	<5	0.1%	<5	0.2%
Nefazodone hydrochloride	B3	14	0.1%	5	0.1%	<5	0.1%	<5	0.1%
Nortriptyline hydrochloride	C	6	0.0%	<5	0.1%	<5	0.1%	0	0.0%

Pregnancy Risk Code: Australian category of risk for the medicine’s use in pregnancy.

* changed from category C to D, September 2005.

The system used in Australia to categorise the risk of drug use in pregnancy is a slightly modified version of the Swedish categorisation (Farmaceutiska Specialiteter i Sverige) and was adopted by the Australian Drug Evaluation Committee (ADEC) in 1989 [14,15]. It includes most of the commonly-used prescription and over-the-counter medicines used in Australia. The categorisations apply only to recommended therapeutic doses in women in the reproductive age group. For pharmaceutical products containing two or more active medicines, the categorisation of the combination is based on the component with the most restrictive categorisation [15]. There are around 950 medicines listed in the ADEC classifications in pregnancy. The antidepressants were classified as B1, B2, B3, C or D for risk for use in pregnancy.

The hospital admission data include a principal diagnosis and up to 20 comorbidities (‘additional diagnoses’) as recorded on the discharge records. The codes are based upon ICD-10-AM [10]. According to the HMDS coding guide that was current during the study, [16] additional diagnoses with the following characteristics need to be coded:

* require therapeutic treatment;

* require performance of a diagnostic procedure;

* increase nursing care and/or monitoring; or,

* may extend the length of stay in hospital.

‘A condition is not routinely coded just because a patient is on ongoing medication treatment of a condition. However, if the medication is altered or adjusted during the episode of care, the condition should be coded.’

We included any hospital admission during pregnancy as well as the birth admission. The codes we used relating to depression are listed in Table 2. These codes are the same as those suggested in the Private Mental Health Alliance [17] and the National Collaborating Centre for Mental Health [18]. Women with any admission that included one of these codes as a principal diagnosis or comorbidity in the record were ascertained as an HMDS case.

**Table 2:** Ascertainment of pregnant women with depression from admissions data, 2002–2005 by ICD-10 codes - 3010 HMDS cases

		**HMD cases**		**HMDS, no PBS**		**HMDS and PBS (AD)**		**HMDS and PBS (not AD)**	
		**N**	**%**	**N**	**%**	**N**	**%**	**N**	**%**
Hospital Morbidity Data System*		**3010**	**100.0%**	**978**	**32.5%**	**899**	**29.9%**	**1133**	**37.6%**
0993	Mental disorders and diseases of the nervous system complicating pregnancy, childbirth and the puerperium	2484	82.5%	791	80.9%	746	83.0%	947	83.6%
F32	Depressive episode	628	20.9%	112	11.5%	399	44.4%	117	10.3%
Z86.5	Personality history of other mental and behavioural disorders	540	17.9%	190	19.4%	151	16.8%	199	17.6%
F33	Recurrent depressive disorder	157	5.2%	37	3.8%	65	7.2%	55	4.9%
F41.2	Mixed anxiety and depressive disorder	97	3.2%	24	2.5%	48	5.3%	25	2.2%
F31.9	Bipolar affective disorder, unspecified	48	1.6%	5	0.5%	27	3.0%	16	1.4%
F31.7	Bipolar affective disorder, currently in remission	9	0.3%	<5	0.4%	<5	0.1%	<5	0.4%
F31.3	Bipolar affective disorder, current episode mild or moderate depression	<5	0.1%	0	0.0%	<5	0.2%	<5	0.1%
F31.4	Bipolar affective disorder, current episode severe depression without psychotic symptoms	<5	0.0%	0	0.0%	<5	0.1%	0	0.0%
F31.6	Bipolar affective disorder, current episode mixed	<5	0.0%	0	0.0%	0	0.0%	<5	0.1%
F31.8	Other bipolar affective disorders	<5	0.1%	0	0.0%	<5	0.2%	<5	0.2%
F31.5	Bipolar affective disorder, current episode severe depression with psychotic symptoms	0	0.0%	0	0.0%	0	0.0%	0	0.0%

AD: antidepressant.

* totals do not equal the sum of the column as women may be diagnosed with more than one disorder or have more than one admission during their pregnancy.

The Australian Bureau of Statistics has released Socio-Economic Indexes for Areas (SEIFA) based on the information collected in the five-yearly Census of Population and Housing. These indexes are widely used measures of relative socio-economic status at a small geographic area level. The indexes rank and identify areas that are relatively more, or less, disadvantaged. They provide contextual information about the area in which a person lives. The indexes have been obtained by principal components analysis which summarises the information from a variety of social and economic variables, calculating weights that will give the best summary for the underlying variables. The categories of variables include income, education, employment, occupation and housing [19].

Three distinct groups were identified: HMDS cases only, PBS cases only, and cases in both datasets. Within the HMDS cases only, two subsets were found: those women dispensed PBS medicines other than antidepressants, and women not dispensed any PBS medicines. These two subsets of HMDS cases are important and we wanted to distinguish between these HMDS groups. The women who were not dispensed any PBS items may be a healthier group; they may be women who had planned their pregnancy and wished to avoid *in utero* exposure to prescription medicines; or, they may have only used medicines which were not captured by the PBS collection (for example: antibiotics dispensed to general patients, complementary medicines, over the counter medicines). Comparisons of demographic, pregnancy, labour and delivery characteristics using MNS data were also made between the women identified with depression using the PBS (any PBS cases) and the remaining women identified with depression using the hospital admissions (HMDS only cases). Odds ratios with 95% confidence intervals (OR; 95% CI) were calculated for all comparisons of prevalence. Student’s *t*-tests were used to compare the means of continuous measures such as maternal age and gestation.

The WADLS uses the Automatch software package [20] with probabilistic matching based upon medical record number, surname, first given name and initial, date of birth, sex and address as the principal matching fields. Missed links have been estimated at 0.11% [21]. The WADLS has been validated previously [21,22] and has been used extensively for health research [23]. All records for this study were also validated internally. For example, sex and dates of birth or death were checked across each source. The researchers received all data in a de-identified form from the WADLS. The datasets were analysed using SAS software, version 9.2 [24]. To fulfil the requirements of ethics committees’ approvals relating to individual privacy, we have not reported cell sizes with less than five study subjects. This project has approval from the Human Research Ethics Committees of The University of Western Australia and the Department of Health WA.

## Results

Based upon hospital admission records and midwives’ notifications, there were 96698 birth events in WA from 2002 to 2005.

### Ascertainment of cases from dispensing records (PBS cases)

At least one antidepressant was dispensed to 4485 (4.6%) women having a birth event with a total of 20879 dispenses of 17 generic medicines (PBS cases): Table 1. There were 38 different forms of antidepressant dispensed with 24 (63%) dispensed to at least 10 pregnant women. Nearly half of all dispenses of an antidepressant were for sertraline hydrochloride (26.5%) or citalopram hydrobromide (22.9%). During the period of this study, 96.1% of the dispenses of antidepressants under the PBS to the pregnant women were as “Restricted Benefit” use which means the medicine can only be prescribed for specific therapeutic uses. These uses are listed in the PBS as ‘major depressive disorders,’ ‘obsessive compulsive disorder,’ and ‘panic disorder where other treatments have failed or are inappropriate.’ This means the women being dispensed the antidepressants under the PBS were confirmed by their clinician as having the need for the therapeutic use required.

### Ascertainment of cases from hospital admission records (HMDS cases)

There were 108088 hospital admissions during pregnancy with 89% of the women having only one admission during their pregnancy and this admission was for the delivery. There were 3010 (3.1%) women with a comorbidity related to depression recorded on their delivery admission, or other admission to hospital during pregnancy (HMDS cases): Table 2. The most common comorbidity codes recorded were ‘O99.3 mental disorders and diseases of the nervous system complicating pregnancy, childbirth and the puerperium’ (82.5%), ‘F32 depressive episode’ (20.9%) and ‘Z86.5 Personal history of other mental and behavioural disorders’ (17.9%).

### Comparison of ascertained cases by dataset

**Figure 1:**
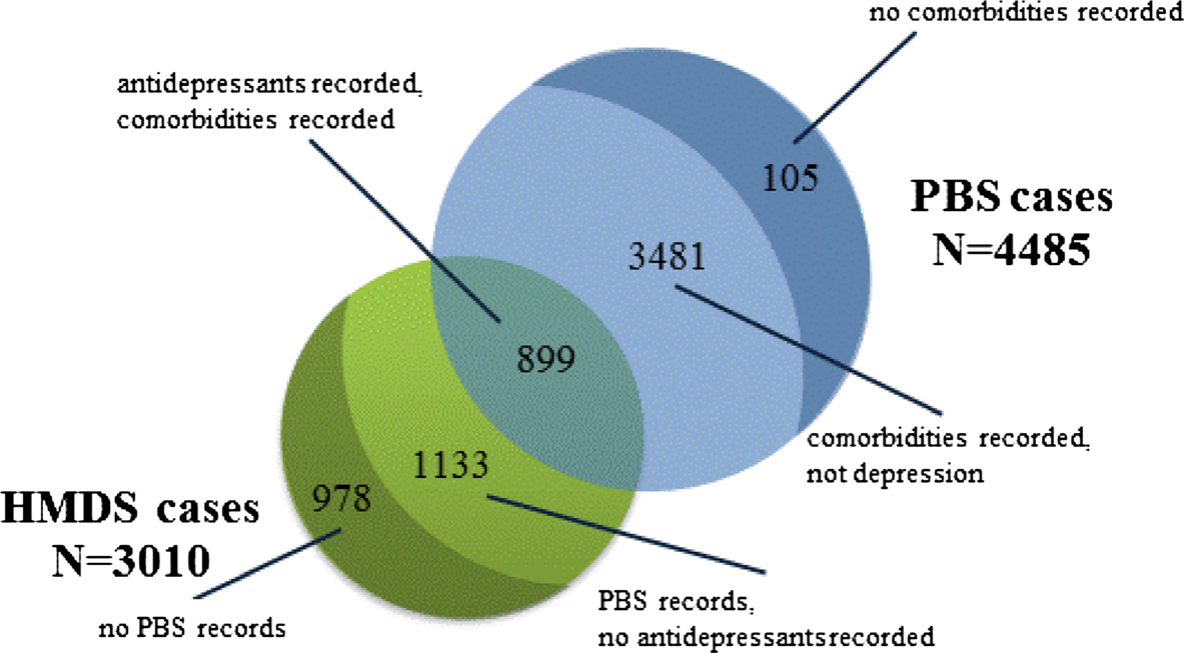
Ascertainment of cases by dataset, N = 6596 pregnancies.

There were a total of 7495 pregnancies identified by either set of records. Using data linkage, we could determine that these cases represented 6596 individual pregnancies (6.8% of 96698 pregnancies). There were 3586 pregnancies that were PBS cases but not HMDS cases (54.4% of all cases) and 2111 pregnancies that were HMDS cases and not PBS cases (32.0% of all cases): Figure 1. Only 899 pregnancies were found in both groups (13.6% of all cases). The areas of the circles in the Venn diagram (Figure 1) are proportional to the number of cases ascertained in each dataset. Using a simple capture-recapture analysis, [25] the estimated total number of cases of depression in the 96698 pregnant women would be 15007 (95% CI: 14274–15741), i.e. 15.5%.

### Other PBS medicines dispensed

Of the 2111 HMDS cases not dispensed an antidepressant but with depression recorded in their hospital admission records, 978 were not dispensed any medicines under the PBS. The remaining 1133 were dispensed a medicine under the PBS but not an antidepressant. The most frequently dispensed medicines were amoxicillin (N = 290, 25.8%), metoclopramide hydrochloride (N = 200, 17.8%) and cephalexin (N = 198, 17.6%).

### Other morbidities recorded for PBS cases

Of the 4485 cases with an antidepressant dispensed (PBS cases), 899 had a comorbidity of depression recorded, 3481 had other comorbidities recorded that were not related to depression, and 105 did not have any comorbidity recorded on their hospital admissions. The most commonly recorded comorbidities for the 3481 cases are listed in Table 3; with ‘Z72 Problems related to lifestyle’ (N = 727, 20.9%) and ‘O70 Perineal laceration during delivery’ (N = 718, 20.6%) being the most common.

**Table 3:** Most frequent comorbidities recorded on hospital admissions, not related to depression, for the 4485 PBS cases

**ICD category**		**N**	**%**
**HMDS comorbidity recorded as depression**		**899**	
**no comorbidity recorded**		**105**	
**HMDS comorbidity recorded, not related to depression**		**3481**	**100.0%**
Z72	Problems related to lifestyle	727	20.9%
O70	Perineal laceration during delivery	718	20.6%
O34	Maternal care for known or suspected abnormality of pelvic organs	608	17.5%
O99	Other maternal diseases classifiable elsewhere but complicating pregnancy, childbirth and the puerperium	583	16.7%
O68	Labour and delivery complicated by fetal stress [distress]	487	14.0%
O80	Single spontaneous delivery	400	11.5%
O72	Postpartum haemorrhage	360	10.3%
O62	Abnormalities of forces of labour	341	9.8%
O92	Other disorders of breast and lactation associated with childbirth	339	9.7%
O36	Maternal care for other known or suspected fetal problems	288	8.3%
O32	Maternal care for known or suspected malpresentation of fetus	276	7.9%
Z29	Need for other prophylactic measures	264	7.6%
O42	Premature rupture of membranes	262	7.5%
O69	Labour and delivery complicated by umbilical cord complications	259	7.4%
O60	Preterm delivery	259	7.4%

### Demographic, pregnancy and delivery characteristics

We investigated the demographic and pregnancy characteristics using data recorded in the MNS: Table 4. We compared the PBS cases (‘any PBS’ cases; N = 4485) with the HMDS cases who were not ascertained in the PBS (‘HMDS only’ cases; N = 2111).

**Table 4:** Demographic and pregnancy, labour and delivery characteristics of all cases, using the midwives’ records

	**HMDS only**		**Any PBS**		**HMDS only**
	**(N = 2111)**		**(N = 4485)**		**vs any PBS**
	**N**	**SD**	**N**	**SD**	**t-test**
Mean maternal age, yrs	29.0	6.1	30.1	5.8	<0.0001
Mean maternal height, cm	164.7	7.1	165.1	6.8	0.0244
Mean gestation, wks	37.7	3.3	38.2	2.3	<0.0001
Mean SEIFA	970.4	99.8	985.1	87.7	<0.0001
	N	%	N	%	OR (95% CI)
Caucasian	1656	78.4	4217	94.0	0.23 (0.19-0.27)
Smoked during pregnancy	743	35.2	1312	29.3	1.32 (118–1.47)
Parity > 1	1584	75.0	3556	79.3	0.79 (0.70-0.89)
Singleton	2056	97.4	4410	98.3	0.64 (0.45-0.90)
**Pregnancy characteristics**					
Preeclampsia	180	8.5	245	5.5	1.61 (1.32-1.97)
Gestational diabetes	97	4.6	210	4.7	0.98 (0.77-1.25)
Essential hypertension	25	1.2	69	1.5	0.77 (0.48-1.22)
Pre-existing diabetes	28	1.3	38	0.8	1.57 (0.96-2.57)
Other medical condition	1189	56.3	2251	50.2	0.78 (0.70-0.87)
CTG ante-partum	972	46.0	1615	36.0	0.66 (0.59-0.73)
CTG intra-partum	1024	48.5	1735	38.7	0.67 (0.60-0.74)
**Labour and delivery characteristics**					
preterm	401	19.0	548	12.2	1.69 (1.46-1.94)
Induced labour	681	32.3	1307	29.1	1.16 (1.04-1.30)
Delivered by an obstetrician	552	26.1	1639	36.5	0.61 (0.55-0.69)
Precipitate delivery	93	4.4	273	6.1	0.71 (0.56-0.91)
Local anaesthetic to perineum	58	2.7	236	5.3	0.51 (0.38-0.68)
Elective Caesarean delivery	337	16.0	857	19.1	0.80 (0.70-0.92)
Emergency Caesarean delivery	408	19.3	663	14.8	1.38 (1.21-1.58)
Fetal distress	408	19.3	705	15.7	1.28 (1.12-1.47)
PPH (> = 500 mls)	468	22.2	508	11.3	2.23 (1.94-2.56)
Pre-labour rupture of membranes	193	9.1	260	5.8	1.64 (1.32-1.99)

In comparison to the women not dispensed an antidepressant but with a comorbidity record indicating depression, the women dispensed an antidepressant were less likely to have a preterm delivery (0.6; 0.5-0.7), and four times more likely to be Caucasian (4.4; 3.7-5.2). These women were more likely to have had a previous pregnancy (1.3; 1.1-1.4) and a singleton birth (1.6; 1.1-2.2). Their delivery was more likely to be attended by an obstetrician (1.6; 1.4-1.8), more likely to be an elective Caesarean (1.2; 1.1-1.4), and less likely to be an emergency Caesarean (0.7; 0.6-0.8). They were more likely to have a precipitate delivery (1.4; 1.1-1.8) and a local anaesthetic to the perineum (2.0; 1.5-2.6). They were less likely to have their pregnancy complicated by preeclampsia (0.6; 0.5-0.8); or to have smoked during their pregnancy (0.8; 0.7-0.9).

## Discussion

The objective of this study was to investigate the differences in ascertainment which may arise between a hospital admission dataset and a dispensing claims dataset, using major depression in pregnancy as an example. Using data linkage, we found records for 6596 pregnancies (6.8% all births) that indicated the mother was dispensed an antidepressant during her pregnancy and/or depression was recorded on her hospital admission records. This proportion is similar to a large Australian study around the same period which reported 8.9% women with an antenatal Edinburgh Postnatal Depression Scale >12 and 5.4% > 14 [9]. Only 899 pregnancies were found in both groups (13.6% of all cases).

80% of women dispensed an antidepressant did not have depression recorded in their hospital records. 70% of women with depression recorded in their hospital admission record were not dispensed an antidepressant. If the true number of women with antenatal depression is around 15% based upon the capture-recapture algorithm, then there are many pregnant women with undiagnosed or undeclared depression in the administrative health datasets. These results are reflected in one of the statements from the National *beyondblue* Perinatal Mental Health program: depression and related difficulties affect around 15 per cent of women during pregnancy and early parenthood, and often goes undetected and untreated [9].

Not all women will continue taking antidepressants whilst they are trying to become pregnant or once they discover they are pregnant for fear of fetal harm. These women may be part of the 978 women ascertained in the HMDS without use of any PBS medicines. This group of HMDS cases may also have been dispensed medicines that are not routinely collected in the PBS. The PBS dataset includes only medicines dispensed under subsidy. Some medicines have a wide range of forms of older medicines that are prescribed but no longer fully subsidised so the number of pregnant women identified as treated would be under-estimated.

The women who were dispensed an antidepressant, the PBS cases, were different in many ways from the HMDS only group (Table 4). From the midwives’ data we found these women were more likely to be Caucasian, have already had at least one delivery, and to have a singleton birth. They were more likely to have a higher socio-economic status. Their delivery was less likely to be preterm, to be induced, or to be an emergency Caesarean section. They were less likely to have a pre-labour rupture of membranes or experience a primary postpartum haemorrhage of ≥ 500 ml. An obstetrician is more likely to have attended the delivery, and more likely to have an elective Caesarean. They were more likely to have a precipitate delivery, and a local anaesthetic to perineum. Many of these characteristics suggest a more medically managed pregnancy and possibly better access to medical services.

The very large proportion of women dispensed an antidepressant but without depression recorded on their hospital records (N = 3586/4485; 80%) is of concern. The comorbidities coded for depression need not routinely be coded just because a patient is on ongoing medication. However, if the medication is altered or adjusted during the episode of care, or the patient requires additional monitoring or nursing care, then the condition should be coded [16]. Women with depression that is well-controlled by medication or other therapies may not inform the hospital clinicians of their depression for a range of reasons; [26,27] or, the clinician may judge that their depression did not require adjustment of therapeutic treatment whilst she was admitted. In either situation, the woman may not be recorded in the HMDS as having depression. In primary health care settings, if depression is not routinely asked about, over 50% of cases are missed, highlighting the need for a systematic approach to perinatal psychosocial assessment [9,28] and a similar situation may occur in the hospital setting. Due to the amount of contact that women have with health care providers during pregnancy, this is the best time to start screening, [29] provided that adequately trained staff are available to supply follow-up services to those identified [30].

As more research into the effects of antidepressants on the newborn is published, [31-35] it is hoped that women will advise their hospital care-givers of their use of antidepressants, particularly third trimester use, so that withdrawal symptoms in the newborn may be managed [36]. Since the time period of this study, the National Perinatal Depression Initiative [37] has been gathering momentum. The Initiative promotes the provision of routine and universal screening for depression for women, once during pregnancy and again about four to six weeks after the birth, by a range of health care professionals including midwives, child and maternal health nurses, general practitioners and Aboriginal health workers – using the Edinburgh Postnatal Depression Scale [38].

No single data source is likely to provide a complete health profile for an individual. For chronic conditions in pregnancy treated with PBS medicines, the hospital admission data do not adequately capture all cases. Data linkage provides a rich resource at a relatively low cost and in a timely manner, than other pregnancy studies in pharmacovigilance whilst maintaining confidentiality. There are several methodological limitations in the current study. The main limitation of using dispensing data relates to whether the medicine was consumed, or consumed as directed, and we have no information in this study for either of these aspects of use. However, by reviewing the repeat dispenses of a medicine, adherence may be inferred. In a previous study of SSRI dispensing patterns, we found 75.4% of the women were dispensed an SSRI in at least two consecutive trimesters, indicating that the women were using the SSRIs [39].

At the time of this study, PBS prescription data was only collected for prescriptions that attracted a Government subsidy. Amendments to the Commonwealth of Australia *National Health Act 1953,* enacted on 23 November 2010, require approved suppliers of Pharmaceutical Benefits Scheme medicines to provide the Australian Government, from 1 April 2012, with data on PBS prescriptions that are priced below the general copayment level (under copayment) [40]. The collection of under copayment information will capture all dispenses of PBS data, thus making the PBS dataset even more valuable for health policy planning, monitoring risk, management protocols, pharmacovigilance and monitoring the quality use of medicines (including polypharmacy) in the community.

## Conclusions

A recent letter by Morton called for more rapid accumulation of evidence regarding the safety of newer medications in pregnancy and lactation as clinicians currently rely on the publication of case reports and case series by single institutions [41]. Kelman et al. advocated the use of data linkage of routinely collected health datasets in Australia for pharmacovigilance in 2007 [42]. Properly applied, automated databases can minimise the cost and reduce the amount of time involved in obtaining information on the effects of marketed medicines [43]. In addition, they can be sufficiently large to study relatively infrequently used medicines. Since medicine exposure is determined from pre-recorded automated data, there is no opportunity for recall bias. Record linkage to a variety of data sources provides the opportunity to control for a wide range of potential confounders [44]. Other major benefits include large sample sizes, generalizability of results, and rapidity of analysis. The data can also be used repeatedly to address a variety of hypotheses or public health questions, and the studies are not intrusive. Data linkage provides a very cost-effective approach to postmarketing surveillance and should provide more timely signals of many adverse events in pregnancy than the methods currently in place.

In order for meaningful results to be extrapolated to specific populations, the limitations of the data and linkage methodology need to be investigated and clarified. This study highlights the limits of case ascertainment within each dataset. It also raises concerns around the reporting of depression in hospital records - whether medical staff are aware that pregnant women are taking antidepressants during their pregnancy, and the consequences this may have for the neonate.

## Competing interests

The authors declare that they have no competing interests.

## Authors’ contributions

LC conceived the study, participated in its design and analysis, and drafted the manuscript. LSS, FJS and CB made substantial contributions to acquisition of data, and provided critical review of the manuscript. All authors read and approved the final manuscript.

## Pre-publication history

The pre-publication history for this paper can be accessed here:

http://www.biomedcentral.com/1471-2393/13/63/prepub

## References

[B1] Alessandri LM Chambers HM Garfield C Vukovich S Read AW Cumulative mortality in children aged 1 to 6 years born in Western Australia from 1980–89 Arch Dis Child 1999 80 1 15 20 10.1136/adc.80.1.15 10325753 PMC1717802

[B2] Brameld KJ Holman CD Bass AJ Codde JP Rouse IL Hospitalisation of the elderly during the last year of life: an application of record linkage in Western Australia 1985–1994 J Epidemiol Community Health 1998 52 11 740 744 10.1136/jech.52.11.740 10396507 PMC1756640

[B3] Hansen M Kurinczuk JJ Bower C Webb S The risk of major birth defects after intracytoplasmic sperm injection and in vitro fertilization N Engl J Med 2002 346 10 725 730 10.1056/NEJMoa010035 11882727

[B4] Spilsbury K Semmens JB Saunders CM Hall SE Holman CD Subsequent surgery after initial breast conserving surgery: a population based study ANZ J Surg 2005 75 5 260 264 10.1111/j.1445-2197.2005.03352.x 15932433

[B5] Holman CD Bass AJ Rosman DL Smith MB Semmens JB Glasson EJ Brook EL Trutwein B Rouse IL Watson CR A decade of data linkage in Western Australia: strategic design, applications and benefits of the WA data linkage system Aust Health Rev 2008 32 4 766 777 10.1071/AH080766 18980573

[B6] Australian Institute of Health and Welfare Mental health services in Australia 2007–08 2010 Canberra: AIHW

[B7] Australian Institute of Health and Welfare Mental health services in Australia 2006–07 2009 Canberra: AIHW

[B8] Australian Institute of Health and Welfare Mental health services - in brief 2011 Canberra: AIHW

[B9] Buist A Bilszta J The beyondblue National Postnatal Screening Program, Prevention and Early Intervention 2001–2005, Final Report. Vol 1: National Screening Program 2006 Melbourne: beyondblue: the national depression initiative

[B10] National Centre for Classification in Health International Statistical Classification of Diseases and Related Health Problems, Tenth Revision, Australian Modification (ICD-10-AM) 1999 Sydney: National Centre for Classification in Health

[B11] Colvin L Slack-Smith L Stanley FJ Bower C Pharmacovigilance in pregnancy using population-based linked datasets Pharmacoepidemiol Drug Saf 2009 18 3 211 225 10.1002/pds.1705 19173342

[B12] Colvin L Slack-Smith L Stanley FJ Bower C Linking a pharmaceutical claims database with a birth defects registry to investigate birth defect rates of suspected teratogens Pharmacoepidemiol Drug Saf 2010 19 11 1137 1150 10.1002/pds.1995 20602344

[B13] Data and Modelling Section, Pharmaceutical Policy and Analysis Branch PBS Expenditure and Prescriptions Twelve Months to 30 June 2006 2006 Canberra: Commonwealth Department of Health and Ageing

[B14] Malm H Martikainen J Klaukka T Neuvonen PJ Prescription of hazardous drugs during pregnancy Drug Saf 2004 27 12 899 908 10.2165/00002018-200427120-00006 15366977

[B15] Prescribing medicines in pregnancy: an Australian categorisation of risk of drug use in pregnancy. 4th edhttp://www.tga.gov.au/hp/medicines-pregnancy.htm

[B16] Department of Health Western Australia Hospital Morbdiity Data System Reference Manual July 2004 2004 Perth: Health Data Collections Branch, Health Information Centre

[B17] Private Mental Health Alliance Private Hospital-based Psychiatric Services 1 July 2010 to 30 June 2011 National Model for the Collection and Analysis of a Minimum Data Set with Outcome Measures for Private Hospital-based Psychiatric Services 2012 Canberra: Centralised Data Management Service

[B18] National Collaborating Centre for Mental Health Depression: the treatment and management of depression in adults (updated edition). Commissioned by the National Institute for Health and Clinical Excellence. National Clinical Practice Guideline 90 2010 London: The British Psychological Society and The Royal College of Psychiatrists

[B19] Australian Bureau of Statistics Socio-Economic Indexes for Areas (SEIFA) - Technical Paper 2006 Information Paper 2001 Census of Population and Housing 2008 Canberra: Commonwealth of Australia

[B20] Automatch. Matchware Technologies Inc Kennebunk, ME, USA

[B21] Holman CD Bass AJ Rouse IL Hobbs MS Population-based linkage of health records in Western Australia: development of a health services research linked database Aust N Z J Public Health 1999 23 5 453 459 10.1111/j.1467-842X.1999.tb01297.x 10575763

[B22] Stanley FJ Croft ML Gibbins J Read AW A population database for maternal and child health research in Western Australia using record linkage Paediatr Perinat Epidemiol 1994 8 4 433 447 10.1111/j.1365-3016.1994.tb00482.x 7870627

[B23] Giles GG Medical record linkage in Australia: this is as good as it gets ANZ J Surg 2005 75 5 259 10.1111/j.1445-2197.2005.03412.x 15932432

[B24] SAS/STAT Copyright © 2009 by SAS Institute Inc Cary, NC, USA

[B25] LaPorte RE McCarty DJ Tull ES Tajima N Counting birds, bees, and NCDs Lancet 1992 339 8791 494 495 1346847 10.1016/0140-6736(92)91103-f

[B26] Yonkers KA Wisner KL Stewart DE Oberlander TF Dell DL Stotland N Ramin S Chaudron L Lockwood C The management of depression during pregnancy: a report from the American Psychiatric Association and the American College of Obstetricians and Gynecologists Gen Hosp Psychiatry 2009 31 5 403 413 10.1016/j.genhosppsych.2009.04.003 19703633 PMC3094693

[B27] Hall P Current considerations of the effects of untreated maternal perinatal depression and the National Perinatal Depression Initiative J Dev Orig Health Dis 2012 3 4 293 295 10.1017/S2040174412000013 25102150

[B28] Johanson R Chapman G Murray D Johnson I Cox J The North Staffordshire Maternity Hospital prospective study of pregnancy-associated depression J Psychosom Obstet Gynaecol 2000 21 2 93 97 10.3109/01674820009075614 10994181

[B29] Austin MP Antenatal screening and early intervention for “perinatal” distress, depression and anxiety: where to from here? Arch Womens Ment Health 2004 7 1 1 6 10.1007/s00737-003-0034-4 14963727

[B30] Pereira AT Bos SC Marques M Maia BR Soares MJ Valente J Gomes AA Macedo A de Azevedo MH The postpartum depression screening scale: is it valid to screen for antenatal depression? Arch Womens Ment Health 2011 14 3 227 238 10.1007/s00737-010-0178-y 20645114

[B31] Adverse Drug Reactions Advisory Committee Maternal SSRI use and neonatal effects Aust Adverse Drug React Bull 2003 22 4 14

[B32] Nordeng H Spigset O Treatment with selective serotonin reuptake inhibitors in the third trimester of pregnancy: effects on the infant Drug Saf 2005 28 7 565 581 10.2165/00002018-200528070-00002 15963005

[B33] Field T Prenatal depression and selective serotonin reuptake inhibitors Int J Neurosci 2010 120 3 163 167 10.3109/00207450802338697 20374082

[B34] Moses-Kolko EL Bogen D Perel J Bregar A Uhl K Levin B Wisner KL Neonatal signs after late in utero exposure to serotonin reuptake inhibitors: literature review and implications for clinical applications JAMA 2005 293 19 2372 2383 10.1001/jama.293.19.2372 15900008

[B35] Chambers CD Johnson KA Dick LM Felix RJ Jones KL Birth outcomes in pregnant women taking fluoxetine N Engl J Med 1996 335 14 1010 1015 10.1056/NEJM199610033351402 8793924

[B36] Field T Diego M Hernandez-Reif M Prenatal depression effects on the fetus and newborn: a review Infant Behav Dev 2006 29 3 445 455 10.1016/j.infbeh.2006.03.003 17138297

[B37] Austin MP Highet N and the Guidelines Expert Advisory Committee Clinical practice guidelines for depression and related disorders – anxiety, bipolar disorder and puerperal psychosis – in the perinatal period. A guideline for primary care health professionals 2011 Melbourne: beyondblue: the national depression initiative

[B38] Cox JL Holden JM Sagovsky R Detection of postnatal depression. Development of the 10-item Edinburgh Postnatal Depression Scale Br J Psychiatry 1987 150 782 786 10.1192/bjp.150.6.782 3651732

[B39] Colvin L Slack-Smith L Stanley FJ Bower C Dispensing patterns and pregnancy outcomes for women dispensed selective serotonin reuptake inhibitors in pregnancy Birth Defects Res A Clin Mol Teratol 2011 91 3 142 152 10.1002/bdra.20773 21381184

[B40] Pharmaceutical Benefits Scheme News Updateshttp://www.pbs.gov.au/info/healthpro/explanatory-notes

[B41] Morton AP A to X: the problem of categorisation of drugs in pregnancy - an Australian perspective Med J Aust 2012 196 3 172 173 22451972 10.5694/mja11.11498

[B42] Kelman CW Pearson SA Day RO Holman CD Kliewer EV Henry DA Evaluating medicines: let's use all the evidence Med J Aust 2007 186 5 249 252 17391088 10.5694/j.1326-5377.2007.tb00883.x

[B43] Stergachis AS Record linkage studies for postmarketing drug surveillance: data quality and validity considerations Drug Intell Clin Pharm 1988 22 2 157 161 3349931 10.1177/106002808802200216

[B44] Libby G MacDonald TM Evans JM Record-linkage methodology for prescribing research J Clin Pharm Ther 2001 26 4 241 246 10.1046/j.1365-2710.2001.00353.x 11493365

